# Use of Point-of-Care Ultrasound by Intensive Care Unit Triage Teams in Evaluating Unstable Patients Outside Intensive Care Units

**DOI:** 10.7759/cureus.49114

**Published:** 2023-11-20

**Authors:** Pranjal B Gupta, Geoffrey Lighthall, Natalie Htet

**Affiliations:** 1 Department of Emergency Medicine, Stanford University, Palo Alto, USA; 2 Department of Anesthesiology, Perioperative and Pain Medicine, Stanford University, Palo Alto, USA; 3 Department of Anesthesia, Veterans Affairs Medical Center, Palo Alto, USA

**Keywords:** point-of-care ultrasound, rapid response, rapid response team (rrt), resuscitation education, ultrasound in critical care

## Abstract

Introduction

Point-of-care ultrasound (POCUS) has become an integral asset in intensive care units (ICUs). However, there is limited literature on the value of POCUS in evaluating deteriorating patients outside the ICU. In this study, we sought to investigate the use and impact of POCUS by ICU triage teams in hospitals outside of the ICU setting.

Methods

ICU triage fellows were provided a portable ultrasound to use as part of their evaluations during consultations and hospital code activations. Fellows were asked to fill out a survey on how ultrasound was used and its impact on patient management. Free-text data such as reason for ultrasound use, views obtained, clinical impressions before and after ultrasound, and clinical actions were recorded. These data were transcribed and categorized electronically.

Results

A total of 51 total resuscitations were documented. The most common reason for ICU triage team evaluation was hypotension (53%, N=27). The most common clinical focus for ultrasound use was cardiac assessment (53%, N=27), followed by volume status assessment (35%, N=18). The most common ultrasound views per encounter obtained were parasternal long (82%, N=42), followed by apical four-chamber view (76%, N=39) and subcostal view (75%, N=38). Out of 38 encounters with clinical impressions documented, 79% (N=30) of pre-ultrasound clinical impressions were confirmed by ultrasound use. Of total encounters, 35% (N=18) had a significant clinical action taken based on ultrasound findings (fluid resuscitation, vasopressor initiation, etc.).

Conclusions

Ultrasound is a valuable tool for patient evaluation in non-ICU wards, especially in confirming clinical impressions and guiding therapeutic actions. Some limitations of this study include reporting bias and incomplete capture of ultrasound use in non-ICU wards.

## Introduction

Point-of-care ultrasound (POCUS) has become an integral asset in the emergency department (ED) and intensive care units (ICUs) [1]. A plethora of literature describes the positive impact of bedside ultrasound use, including improving hands-on time during cardiac arrest [2], improving diagnostic accuracy and guiding clinical management in critically ill patients [3-6], and enhancing procedural efficiency [4]. Careful use of POCUS is important, as there is also evidence that POCUS prolongs pulse checks during cardiac arrest resuscitation [7]; in addition, minimizing pulse check duration with educational interventions is important [8].

However, the use of bedside ultrasound outside critical care areas, such as in the hospital wards, is lagging behind [9]. There is little literature on the value of POCUS in evaluating patients in the wards, specifically in ward emergencies [10-12]. POCUS can have a high impact on rapid response systems (RRS), also known interchangeably as rapid response teams or medical emergency team, which is a dedicated multidisciplinary team of nurses, physicians, technicians, pharmacists, and respiratory therapists deployed to ward emergencies [10,13].

In this study, we sought to investigate the use and impact of POCUS by ICU triage teams in hospitals outside of the ICU. Our aim is to further contribute to the growing literature of ward-based emergencies and ultrasound use to understand how clinical decision-making is affected by ultrasound use, as well as the reasons POCUS is being used. In particular, we focus on ultrasound use by ICU triage teams, led by a dedicated ICU fellow physician (ICU triage), with a portable bedside ultrasound in the evaluation of critically ill patients during ICU consultations, rapid response alerts, “Code Sepsis,” and “Code Blue” alerts on the ward.

## Materials and methods

The study was conducted at two hospitals (Stanford Hospital and Veterans Affairs Palo Alto Health System) within a multidisciplinary critical care fellowship. Fellows were trained on POCUS through a curriculum consisting of echocardiograms, lung ultrasound, deep vein thrombosis (DVT) ultrasound, and Extended Focused Assessment with Sonography in Trauma (eFAST) through monthly lectures and scanning shifts with ultrasound technicians and attending physicians. The fellows underwent 4 hours of a scanning shift at the beginning of fellowship and individually worked toward acquiring 50 echocardiograms, 10 lung ultrasounds, 10 DVT, and 10 eFAST scans to graduate from fellowship. The images acquired by fellows were reviewed by the dedicated critical care faculty with ultrasound training on a routine basis prior to graduation.

At Stanford Hospital, the ICU triage fellow responds to consultations, rapid response alerts, “Code Sepsis,” or “Code Blue” activations 24/7. At the Veteran Affairs Palo Alto Health System, the ICU triage fellow has a similar role and is available to respond to emergencies within 20 minutes. ICU fellow physicians had access to several ultrasound machines in the hospital (SonoSite Edge, SonoSite MicroMaxx, GE Venue Go) and were also given handheld pocket ultrasounds (Vscan, Philips Lumify) to use in their evaluations. Fellows were encouraged to use POCUS when a consultation, rapid response alert, “Code Sepsis,” or “Code Blue” occurred and to subsequently document the setting of ultrasound use, reason for ultrasound use, ultrasound views obtained, clinical impression prior to ultrasound use, and clinical impression after ultrasound use on pre-made index cards. Fellows had the autonomy to apply POCUS on ward emergencies, with support of critical care attendings, but the images were not reviewed by ultrasound attendings immediately. POCUS was not a mandated equipment to respond to these emergencies. Identifying patient information, such as name, date of birth, or medical record number, were not collected on the index cards.. The study aimed to reflect and understand POCUS use in ICU triage and initial resuscitation phase in the diagnosis and management of undifferentiated patients. This study was approved by the Stanford Human Subjects Committee and Veterans Affairs Institutional Review Board.

Descriptive notecards were collected and coded into Microsoft Excel for analysis. Subjective comments were categorized based on whether the pre-ultrasound impression was confirmed or changed and whether any significant clinical action occurred as a result of ultrasound use. Standard descriptive statistics were calculated and analyzed using Stata [14].

## Results

A total of 51 clinical encounters using portable ultrasound were documented by 14 different ICU fellow physicians between 2014 and 2018. The clinical context of the encounters is shown in Table 1. ICU consultation was the most frequent source of collected data (63%, N=32), followed by rapid response alerts (24%, N=12) and “Code Blue” events (14%, N=7). Locations were recorded at 46 instances; 57% (N=26) of locations were hospital wards, while 20% (N=9) were in the ICU and 17% (N=8) were in the ED. Also, 52% (N=27) of the encounters focused on cardiac function assessment, while 35% (N=18) focused on volume status assessment. A variety of ultrasound views were obtained, including echocardiographic views: parasternal long, parasternal short, subcostal, apical four-chamber, apical two-chamber, and inferior vena cava views, as well as thoracic, deep venous, and Focused Assessment with Sonography for Trauma (FAST) views (Table 2).

**Table 1:** Clinical context of ICU triage encounter ICU, intensive care unit

Context of ICU triage	N (%)
ICU consultation	32 (63%)
Rapid response	12 (24%)
“Code Blue”	7 (14%)

**Table 2:** Views obtained during POCUS PSL, parasternal long axis; PSS, parasternal short axis; DVT, deep vein thrombosis; eFAST, Extended Focused Assessment with Sonography in Trauma; POCUS, point-of-care ultrasound

Views obtained	Number	Percentage
PSL	42	82.4
PSS	37	72.6
Subcostal	38	74.5
Apical four-chamber	39	76.5
Apical two-chamber	9	17.6
Lung	9	17.6
DVT	2	3.92
eFAST	2	3.92

Of the 51 encounters, 38 had documented clinical impressions before and after ultrasound use. In addition, 79% of clinical impressions were confirmed with ultrasound (N=30, out of 38), while the other 21% of clinical impressions changed with ultrasound (N=8, out of 38) (Table 3). When assessing cardiac function, 74% of impressions were confirmed (N=14 out of 19). When assessing volume status, 80% of impressions were confirmed (N=12 out of 15).

**Table 3:** Evaluation of clinical impression and clinical action taken with POCUS DVT, deep vein thrombosis; FAST, Focused Assessment with Sonography in Trauma; POCUS, point-of-care ultrasound

Clinical query evaluated with POCUS	Clinical impression confirmed with POCUS, % (n/total)	Clinical action taken after POCUS, % (n/total)
Cardiac assessment	74% (14/19)	36% (9/27)
Volume status	80% (12/15)	59% (10/18)
DVT	100% (1/1)	100% (1/1)
FAST	n/a (0/0)	0% (0/1)
Pneumothorax	100% (2/2)	0% (0/3)
Pleural effusion	100% (1/1)	0% (0/1)
Total	79% (30/38)	35% (18/51)

Of all encounters, 35% of encounters had a clinical action taken after ultrasound findings (N=18). Clinical actions were fluid resuscitation (67%, N=12, out of 18), initiation of medications such as vasopressors or tissue plasminogen activator (17%, N=3, out of 18), or decision regarding appropriate disposition of the patient (17%, N=3, out of 18). Clinical action was taken 33% (N=9, out of 27) of the time when evaluating cardiac function and 44% (N=10, out of 18) of the time when evaluating volume assessment. A sampling of subjective comments regarding the use of ultrasound is given in Table 4.

**Table 4:** Narrative excerpts of transcriptions of the handwritten comments regarding ultrasound usage CHF, congestive heart failure; FFP, fresh frozen plasma; IABP, intra-aortic balloon pump; IVC, inferior vena cava; PE, pulmonary embolism; POCUS, point-of-care ultrasound; RV, right ventricle; tPA, tissue plasminogen activator; VAD, ventricular assist device

Narrative excerpts	
Before POCUS	After POCUS
"Vasovagal, normal heart function"	"…Normal contraction of LV on echo, US confirmed clinical suspicion"
"Myocardial ischemia"	"New wall motion abnormality, myocardial ischemia, study escalated management and need to decide about VAD, IABP, etc."
"Fluid overload"	"Multiple B lines in lungs -> fluid overload"
"Septic shock, hypovolemia"	"IVC collapsible, OK cardiac function, started IV bolus"
"Suspect a massive PE"	"RV was dilated, tPA was given"
"RV mild dysfunction"	"RV is OK. Hypotensive due to low volume"
"Patient is volume overloaded"	"Confirmed clinical suspicion. Patient is very much volume overloaded"
"Sepsis and concern for volume overload"	"Happy RV + collapsible IVC (1.2cm -> flat), we gave all the fluid!"
"Unsure possible PE"	"No RV Strain, No septal bowing, RV function OK, No indication for tPA"
"Possible CHF, needs lots of volume"	"Looked like he could handle 4+ units of FFP"
"Volume down, hypovolemic"	"Same as pre-echo suspicion"
"Sepsis shock hypovolemia"	"IVC collapsible, ok cardiac function, started IV bolus"

## Discussion

This was a descriptive study designed to evaluate the pattern of POCUS use outside ICU. Our data suggest that POCUS may have an important role in confirming clinical impressions and influencing clinical actions in the management of unstable patients. POCUS continues to be adapted into clinical practice across all areas of the hospital, and this study adds to the growing literature regarding this expanded POCUS application. Technological advancement and easy accessibility have allowed POCUS to emerge as an aide to traditional diagnostic and management techniques in medicine [15,16]. Several studies have been published that highlight an improved diagnostic accuracy and reduced time to diagnosis with POCUS, but there are conflicting data to highlight a difference in patient outcomes [16-19]. A major multi-center randomized controlled trial on POCUS use in undifferentiated hypotensive patients in the ED did not identify benefits for survival, length of stay, rates of CT scanning, inotrope use, or fluid administration compared to standard of care [20]. Some other studies, on the contrary, have proved a change in clinical outcomes [21,22]. Regardless, POCUS use has been argued as a tool to augment the understanding of physiology and change in clinical decision-making [23].

This study has several limiting factors. The study has a small number of participants, and not all ultrasound uses by ICU triage teams were recorded. As such, although these subjective trends in the data are informative, it is difficult to make broad objective claims. Furthermore, these data are subject to significant reporting bias, as we did not ensure that an equal representation of ICU triage fellow physicians was sampled. The number of ultrasounds completed in the study period was several-fold higher than the number reported here. Physicians who were more comfortable with POCUS may have been more likely to participate in the study, and those physicians may have a higher likelihood of using POCUS strategically to impact clinical decision-making. Although fellow physicians were trained on POCUS, there was no validation at the beginning of the study to confirm that they were at similar competency in the use of ultrasound. Furthermore, given the subjective nature of narrative comments, transcribing them into categorical values is subject to interpretation error. We did not ensure that pre-POCUS impression was documented by the ICU fellow physician prior to the scan being done; therefore, the study is subject to recall bias. In addition, there is no way to validate that the clinical action taken by fellows was the correct action. Fellows were given autonomy to exercise their clinical judgment, and how POCUS impacts their judgment was simply recorded. Finally, although we wanted to only evaluate POCUS use in wards by ICU triage teams, some POCUS was performed in the ICU and the ED. However, all the patients were in the initial undifferentiated diagnosis and management stage of resuscitation, indicating that POCUS may be useful and an integral part of rapid response/triage team workflow.

## Conclusions

Overall, this study highlights the future of ultrasound use in medicine and sparks several intriguing discussions. Ultrasound has slowly integrated into clinical practice, and several medical schools have incorporated ultrasound into the curriculum of new medical students. Similarly, ultrasound use has been adopted into nurse-driven protocols. A majority of hospitals have structured rapid response teams with varying members with a lead nurse or physician.

Clinicians should consider the benefit of including a portable ultrasound to standard tools and protocols for the determination and management of deteriorating patients in RRS. A multispecialty collaboration at the institutional level is necessary to facilitate the organization of the curriculum, training of faculty, and quality assurance. Additionally, an expert panel and consensus guideline by professional organizations at the national level are needed to standardize POCUS training as POCUS may become a mandated tool in the future.
